# Cu transporter protein CrpF protects against Cu-induced toxicity in *Fusarium oxysporum*

**DOI:** 10.1080/21505594.2020.1809324

**Published:** 2020-08-30

**Authors:** Damaris Lorenzo-Gutiérrez, Lucía Gómez-Gil, Josep Guarro, M. Isabel G. Roncero, Javier Capilla, Loida López-Fernández

**Affiliations:** a Unitat de Microbiologia, Facultat de Medicina i Ciències de la Salut and Institut d’Investigació Sanitària Pere Virgili (IISPV), Universitat Rovira i Virgili, Reus, Spain; b Departamento de Genetica, Facultad de Ciencias and Campus De Excelencia Internacional Agroalimentario ceiA3, Universidad de Cordoba, Cordoba, Spain

**Keywords:** Copper (Cu) transport, Cu homeostasis, fungal pathogenesis, P_IB_-type ATPase, Crp

## Abstract

Cu is an essential trace element for cell growth and proliferation. However, excess of Cu accumulation leads to cellular toxicity. Thus, precise and tight regulation of Cu homeostasis processes, including transport, delivery, storage, detoxification, and efflux machineries, is required. Moreover, the maintenance of Cu homeostasis is critical for the survival and virulence of fungal pathogens. Cu homeostasis has been extensively studied in mammals, bacteria, and yeast, but it has not yet been well documented in filamentous fungi. In the present work, we investigated Cu tolerance in the filamentous fungus *Fusarium oxysporum* by analysing the Cu transporter coding gene *crpF*, previously studied in *Aspergillus fumigatus*. The expression studies demonstrated that *crpF* is upregulated in the presence of Cu and its deletion leads to severe sensitivity to low levels of CuSO_4_ in *F. oxysporum*. Targeted deletion of *crpF* did not significantly alter the resistance of the fungus to macrophage killing, nor its pathogenic behaviour on the tomato plants. However, the targeted deletion mutant Δ*crpF* showed increased virulence in a murine model of systemic infection compared to wild-type strain (wt).

## Introduction

Within prokaryotic and eukaryotic cells, heavy metals such as Cu, Zn, or Fe, among others, have essential roles as cofactors in several enzymatic reactions [1,2]. However, elevated concentrations of these metals result highly toxic due to substitution of specific enzymatic cofactors leading to biochemical alterations [3–5]. Excessive accumulation of transition metals, such as Cu, also promotes the generation of hydroxyl radicals, which induces severe cellular damage. Accordingly, biological systems have evolved strategies for the maintenance of metal homeostasis finely controlled within the cells [6–8].

Acquisition and distribution of Cu in eukaryotes have been studied in the yeast model *Saccharomyces cerevisiae* [1,2,9,10]. Although many of the homeostatic mechanisms involved in the acquisition, use and regulation of Cu are conserved in many species, different fungi have evolved distinct mechanisms that enable them to adjust to particular environments [10].

Several authors have demonstrated that fungi can adapt to environments where metals range from limiting to excessive concentrations, this latter fact due to industry contaminants, inorganic fertilizers, and pesticides [11,12]. In addition, pathogenic fungi have to cope with toxic conditions during infectious processes in mammals, since their hosts use Cu compartmentalization within macrophages to deliver toxic Cu during fungal infections as a defence mechanism [13–15].

Nevertheless, fungi are known to harbour different features concerning Cu detoxification and homeostasis mechanisms. While some use metallothioneins as main defence strategy [16], others show export proteins to regulate Cu levels [17].

Cu-exporting ATPases belong to a superfamily of proteins known as P_1B_-type heavy-metal ATPases (HMA) with ion pump functions that transport specific ions across the cell membrane against a concentration gradient using the energy stored as ATP [18,19]. Cu-exporting ATPases display eight conserved functional domains and function by catalysing the phosphorylation of a key conserved aspartate residue within their catalytic site, followed by hydrolytic cleavage of ATP and the displacement of bound ions. P_IB_-type ATPases are membrane-associated proteins that traffic among Endoplasmic Reticulum (ER), Golgi and plasma membrane changing their location in response to Cu concentrations and involved in Cu^+^, Ag^+^, Zn^2+^, Cd^2+^, or Pb^2+^ pumping out the cell [20–22].

Cu-exporting ATPases have been studied in many fungal pathogens, demonstrating their relevance in Cu homeostasis and pathogenesis. This is the case of the Cu-transporting P_IB_-type ATPase CaCrp1 in *Candida albicans*, which is inducible by Cu and confers resistance to toxic levels of this metal being also required for full fungal virulence [16,22]. Particularly, Ca*Crpa1* has been shown to be essential for fungal survival in the presence of low Cu concentrations in acidic and anaerobic environments, such as infection conditions [22].

In *A. fumigatus*, the Cu-exporting CrpA has been shown to confer resistance to Cu, as the defective mutant Δ*crpA* is hypersensitive to this metal ion. Also, CrpA is considered a defence mechanism against host during infection, as Δ*crpA* displayed reduced viability in murine macrophages infection and decreased virulence in a non-neutropenic invasive aspergillosis murine model [17]. Similarly, in *A. nidulans crpA* deletion led to severe sensitivity to toxic Cu and morphological growth defects [20]. Exceptionally, the genome of *Aspergillus flavus* contains two *crp* genes: *crpA* and *crpB*. This gene duplication is considered responsible for the greater Cu tolerance displayed by this fungus, being able to grow at higher concentrations of Cu than those tolerated by *A. fumigatus* and other *Aspergillus* spp. [23].

Although several studies have attempted to understand metal homeostasis in *C. albicans* and *Aspergillus* species, there is a lack of knowledge in other important fungal pathogens. Thus, the present work has focused on *F. oxysporum*, since this species is a multi-host model organism [24] showing multidrug-resistance able to cause severe disease in human beings and great losses in crops. Recently we have reported the identification of a metallothionein (*mt1*) in *F. oxysporum* which seems to be activated specifically by Zn and is involved in defence against Cu and reactive oxygen species (ROS) [25]. The aim of the present study was to evaluate the contribution of Cu-exporting ATPases in metal homeostasis and virulence in this fungus. By *in silico* search in the genome of *F. oxysporum*, we have identified a *crpF* gene, coding for Cu-exporting ATPase. We have studied gene expression and function of *crpF* gene by generation of a knockout strain and its subsequent characterization. Expression analysis of *crpF* and other metal-related genes (*mt1* and *aceA*) and stress response genes (*prx* and *gapdh*) were evaluated in the presence of different heavy metals in wt and the mutant strain. This work provides a better understanding of the function of Cu-exporting ATPase in *F. oxysporum* and helps to complete the picture of the role of Cu in fungal pathogenesis.

## Material and methods

### Fungal strains and culture conditions

*F. oxysporum* f.sp *lycopersici* wild-type strain 4287 was used in all experiments. The fungus was stored at −80°C with glycerol as microconidial suspension. For extraction of genomic DNA (gDNA) from mycelia and microconidia production, cultures were grown in potato dextrose broth (PDB) with shaking at 170 rpm, as previously described [26]. Determination of the minimal inhibitory concentration (MIC) of Cd, Cu, and Zn was performed in 96-well microplates containing Synthetic Medium (SM) (0.2 g L^−1^ MgSO_4_ · 7H_2_O, 0.4 g L^−1^ KH_2_PO_4_, 0.2 g L^−1^ KCl, 1 g L^−1^ NH_4_NO_2_, 0.01 g L^−1^ FeSO_4_, 0.01 g L^−1^ ZnSO_4_, 0.01 g L^−1^ MnSO_4_, 10 g L^−1^ glucose, and 15 g L^−1^ agar) supplemented with CdCl_2_ (0.1, 0.15, 0.25, and 0.3 mM), ZnCl_2_ (15, 20, 25, and 30 mM) or CuSO_4_ (0.3, 0.4, 1, or 1.5 mM). Control wells consisted on SM without metals addition. Microplates were inoculated with 30 µL of a suspension containing 10^6^ spores/mL and incubated for 10 days at 28°C.

For phenotypical characterization of colony growth, water droplets containing 10^3^, 5 × 10^2^ or 10^2^ freshly microconidia obtained from a 3–4 days old culture were spotted onto solid Synthetic Medium (SM) supplemented with 0.15 mM CdCl_2_, 20 mM ZnCl_2_ or 0.4 mM CuSO_4_. Plates were incubated at 28°C for 3–15 days before being photographed. For infection experiments, freshly microconidia were obtained from a 3–4 days old culture. For analysis of gene expression, freshly obtained microconidia were germinated in PDB at 28°C and 170 rpm for 12 h and transferred to liquid SM medium supplemented with 0.1 mM CdCl_2_, 0.175 mM CuSO_4_ or 7.5 mM ZnCl_2_, and incubated for other 6 h. Then, mycelium was harvested by filtration, washed with distilled water, frozen in liquid nitrogen, and stored at – 80°C until its use for RNA and DNA extractions.

### Nucleic acid manipulations

Total RNA and genomic DNA (gDNA) were extracted from *F. oxysporum* ground mycelium in liquid nitrogen with mortar and pestle. Total gDNA was isolated according to previously reported protocol [27] and total RNA was extracted with Trizol Reagent (Invitrogen Life Technologies, CA, USA) [28]. DNA and RNA extractions were resuspended in DNase and RNAse-free double-distilled water and their quality determined by running aliquots in RedSafe-stained agarose gels and analysed in a Nanodrop2000™ spectrophotometer (Thermo Fisher). Southern analyses and probe labelling were carried out as described previously [26] using the non-isotopic digoxigenin-labelling kit (Roche, IN, USA).

### Bioinformatic

BLASTP searches were performed in order to find orthologues proteins of *A. fumigatus* CrpA, PcaA, and CptA (Ccc2) in *F. oxysporum* and determine sequence similarity at NCBI (http://www.ncbi.nlm.nih.gov/blast) and *F. oxysporum* genome database (https://genome.jgi.doe.gov/Fusox1/Fusox1.home.html). For phylogenetic studies, amino acid sequences were aligned using CLUSTALW algorithm [29], followed by 1,000 nonparametric bootstrap analysis in MEGA v. 6.06 software [30]. A phylogenetic tree was built from the retrieved hits from BLASTp search, in which the most representative species of each genus were included. Phylogram was constructed using the Neighbour-Joining method.

Transmembrane domains were predicted using the web-based software Protter (http://wlab.ethz.ch/protter/start/).

### Targeted gene deletion and complementation

Targeted gene replacement of entire coding region of the *crpF* (FOXG_03265) was performed using the split-marker technique [31]. Briefly, two overlapping constructs were generated by fusion PCR using Expand High Fidelity PCR System (Roche Diagnostics) to delete *crpF* gene. The 5´and 3ʹ genomic flanking sequences of *crpF* were obtained by PCR amplifications of wild-type gDNA. The promoter fragment of 896 bp, obtained by amplification with the specific primer pair crp-F2 n and crpM13F-R1, was fused to the 3′ end of the hygromycin resistance cassette (Hyg^R^) [32] and amplified using primers M13 F and hyg-G to generate the first construct that contains a partial sequence of Hyg^R^ cassette (approximately 75%) (Table 1). The second construct contained the terminator region of *crpF* of 868 pb, obtained by amplification with the specific primer pair crp-M13R-F3 and crp-R3 n, fused to the 5′ end of the Hyg^R^ cassette, amplified using the primer pair M13 R and hyg-Y which results in construct that contains a partial sequence of Hyg^R^ cassette (approximately 75%). These two overlapping constructs were used to transform protoplasts from the *F. oxysporum* wild-type strain (Figure 3(a)), as reported previously [26]. Protoplasts of *F. oxysporum* were generated using 10% (w/vol) of Extralyse (Laffort, Bordeaux, France) for digestion of cell walls [26]. The resulting Hyg^R^ transformants were initially screened by PCR (Figure 3(b)), and Southern blot analyses were then used to identify homologous recombination events.

**Table 1.** Oligonucleotides used in this study.

Name	Sequence (5ʹ – > 3ʹ)	Position to ATG	Experimental use
CrpF-F1	GGTAGTTTTGCTTCTGCTGTT	−1021 (s)	Δ*crpF* construction, complementation
CrpF-M13 F-R1	*gtcgtgactgggaaaaccctggcg*GCGTTGTGTGTTGTGATGAAA	− 43 (as)	Δ*crpF* construction, probe
CrpF-M13 R-F3	*tcctgtgtgaaattgttatccgct*TTAGGAATGGCGGTAGTGGT	+ 3341 (s)	Δ*crpF* construction
CrpF-R4 n	ACCGCAAACAGTCAAATCCTTC	+ 3848 (as)	Δ*crpF* construction
CrpF-R2	AGTGAATGTGTAAGCCAGTGT	+ 4341 (as)	Δ*crpF* construction, complementation
CrpA-F2 n	TGGTCCCGTTTCTCAAGGTG	− 918 (s)	Δ*crpF* construction, probe
CrpF-F5	CCGTTGCTCTGCCGTATCTT	− 556 (s)	Δ*crpF* construction
CrpF-R6	TCCTCTCCTCTCCTCTCCAC	+ 4610 (as)	Δ*crpF* construction
CrpF-F4	TGATTCGCTCCCTCTTACACG	+ 73 (s)	RT-PCR
CrpF-R4	GCTACTCTCCCCGTCAACCT	+ 280 (as)	RT-PCR
act-9	GCGGTTACACTTTCTCCACCA	+587 (s)	RT-PCR
act-10	TTGAAGGTGGTGACATGGATAC	+818 (as)	RT-PCR
mt1-12	TGCTCATGCGGTCAAAAGTCC	+150 (s)	RT-PCR
mt1-13	CCATCAGCAGCCTTCTCGCA	+396 (as)	RT-PCR
G3PDH-F1	ACCACCGTCCACTCCTACAC	+139 (s)	RT-PCR
G3PDH-R2	GATCTGGTCGTAAGAAGCACC	+367 (as)	RT-PCR
Peroxi-F1	GCCTTCACCATCCGATCCGT	+438(s)	RT-PCR
Peroxi-R2	GGCTTGACGATGCGGAACTC	+666(as)	RT-PCR
hyg-G	CGTTGCAAGACCTGCCTGAA	-	Hyg^R^ cassette
hyg-Y	GGATGCCTCCGCTCGAAGTA	-	Hyg^R^ cassette
M13 F	CGCCAGGGTTTTCCCAGTCACGAC	-	Hyg^R^ cassette, Nat^R^ cassette
M13 R	AGCGGATAACAATTTCACACAGGA	-	Hyg^R^ cassette, Nat^R^ cassette
M13 F2	GCATTCTGGGTAAACGACTC	-	Hyg^R^ cassette, PCR verification
M13 R2	CGAGACCTAATACAGCCCC	-	Hyg^R^ cassette

Italics and lower case indicate nucleotide sequences added for cloning purposes. Positions are referred to the start codon, (+) downstream or (-) upstream of ATG. Orientation is indicated, (s) sense, (as) antisense.

For complementation of the *ΔcrpF* mutant, a 3.3 kb fragment containing the complete *crpF* gene (including promoter and terminator regions) was amplified from gDNA using primers crp-F1 and crp-R2. The amplified fragment was used to co-transform protoplast of the *ΔcrpF* with the 1.4 kb nourseothricin resistance cassette (Nat^R^), amplified from plasmid pDNat1 [33] using universal primers M13 F and M13 R. The resulting Nat^R^ transformants were screened by Southern analyses to confirm integration of *mt1* gene.

### Expression analyses

Total RNA (1 µg) treated with DNase I (Thermo Fisher) was reverse-transcribed into first-strand-complemented DNA (cDNA) with iScriptTM cDNA Synthesis Kit (Bio-Rad Laboratories, Inc. Hercules, CA, USA) using a poly-dT antisense primer, according to the manufacturer’s instructions. Amplification of *mt1, crpF* (FOXG_03265), *aceA* (FOXG_03428), *gapdh* (FOXG_08006), *prx* (FOXG_15430) and the housekeeping gene *act1* (FOXG_01569) [34] was performed from cDNA using specific primers designed on Oligo 7 software (Molecular Biology Insights, Inc., Colorado Springs, CO, USA) (Table 1). Endpoint PCRs were performed with (Biotaq^TM^, Bioline) as follows: 95°C for 5 min, 30 cycles of 35 s at 94°C, 30 s at 60°C and 30 s at 72°C, followed by a final elongation of 7 min at 72°C. The PCR products were visualized by electrophoresis on 1.5% RedSafe stained agarose gels.

### Cu intracellular quantification

Intracellular determination of Cu content in wt, Δ*crpF* and Δ*crpF^c^* strains was performed as previously described [35,36]. Briefly, microconidia were obtained from 3 to 4 days old cultures (28ºC and 170 rpm) and germinated in PDB (same conditions) for 12 h. Then, germlings were transferred to liquid SM with or without 100 µM CuSO_4_ and incubated for 6 or 24 hours at 28°C and 170 rpm for biomass obtention. The mycelium was harvested by filtration, washed with distilled water containing 10 mM of citric acid in 0.5% (w/v) NaCl in order to remove Cu ions adsorbed on the cell surface. *F. oxysporum* mycelium was frozen in liquid nitrogen, ground, and dried in a laboratory oven. For each measurement 40 mg of dry biomass was re-suspended in 300 μL of 20% (w/v) trichloroacetic acid (TCA), transferred to a 2 mL screw cap tube containing 100 μL of glass microbeads and subjected to mechanical lysis by three cycles of 20 seconds at maximum speed with a Fast Prep® – FP120 instrument (Thermo Savant, Holbrook, NY). The crude extract was clarified by centrifugation at 10,000 g for 10 min and the supernatant transferred to a new tube. The clarified crude extract was diluted in 500 μL of deionized water and added to 100 μL of 1% (w/v) ascorbic acid and 400 μL of BCA reagent (0.006% (w/v) 2,2ʹ-biquinoline-4,4ʹ-dicarboxylic acid disodium salt hydrate) Sigma-Aldrich (Saint Louis, USA), 3.6% (w/v) NaOH, 15.6% (w/v) Hepes. After 2 min of incubation at room temperature, absorbance was recorded at 354 nm using UV with a Nanodrop 2000™ spectrophotometer (Thermo Fisher). The amount of Cu was calculated using the Beer–Lambert law: Absorbance (A) = ε (molar extinction coefficient) x c (molar concentration) x l (path length). Concentrations were calculated using the molar extinction coefficient of BCA-Cu(I) complex at 354 nm (4.6 x 10^4^ litre mol^−1^ cm^−1^) [36]. Conversion of BCA-Cu(I) complex in mol/L to µg of Cu was done using the molecular mass of BCA-Cu(I) and the atomic mass of Cu. The amount of Cu was referred as µg Cu per mg of dry biomass.

### Infection assays in plant, murine and macrophages cell line

Tomato root inoculation assays were performed as previously described [26], using 2-week-old tomato seedlings (cultivar Monika, seeds kindly provided by Syngenta, Spain). Tomato roots were submerged in a spores suspension containing 5 × 10^6^ spores mL^−1^ of *F. oxysporum* for 30 min and then planted in vermiculite and maintained in a growth chamber. Ten plants were used for each assayed strain, i.e. wt, Δ*crpF* and Δ*crpF^c^*. Severity of disease symptoms and plant survival was recorded daily for 30 days as previously described [37]. Experiments were repeated three times with similar results. Data presented are from one representative experiment.

For mice infection, four-weeks-old male OF1 mice weighing 28–30 g were used (Charles River, Criffa SA, Barcelona, Spain). Groups of 8 animals were immunosuppressed by intraperitoneal administration of 200 mg/kg of cyclophosphamide (Genoxal; Laboratories Funk SA, Barcelona, Spain), beginning 2 days prior infection and then every 5 days until the end of the experiment. Mice were inoculated intravenously (i.v.) through the lateral tail vein, with a suspension of 10^7^ or 2 × 10^7^ microconidia per animal in 0.2 mL of sterile saline solution. Virulence was evaluated by survival through 15 days and by fungal load quantification in kidneys and lungs 7 days after infection [38].

Adherent macrophage-like cell line J774.1 was maintained in tissue culture flasks at 37°C with 5% CO_2_ in Dulbecco’s Modified Eagle medium (DMEM; Biowest, MO, USA) with L-glutamine supplemented with 10% (v/v) fetal bovine serum and 1% penicillin/streptomycin. For killing assays macrophages were scraped from the tissue culture flask and transferred to 6-wells culture plate in DMEM and incubated overnight at 37°C, 5% CO_2_ for 20–24 h prior to the infection [39]. Infection was performed by adding 1.6 × 10^6^ *F. oxysporum* germlings (MOI 1) obtained from 8 h cultures at 37°C and 120 rpm in DMEM. The infection assay was maintained for 6 h at 37ºC and 5% CO_2_. Then, wells were washed twice with 1X PBS in aim to remove non-phagocytosed germlings. Subsequently, cells were lysed by cold water osmotic shock and plated onto PDA for fungal viability. Experiments were done by triplicate with duplicate wells per sample.

### Statistical analyses

Mean survival times on animal and plant infection were estimated from Kaplan-Meier survival curves and compared using the log-rank test. The tissue burden studies results were analysed using the Mann–Whitney U test and the t-test by Significance of transcriptional levels and survival rates from the macrophage-killing assay were determined using a paired t-test. All statistical analyses were performed in Graph Pad Prism 6.0 (GraphPad Software, CA, USA) for Microsoft Windows. *P* values ≤ 0.05 were considered statistically significant.

## Results

### *Identification of Cu-transporter encoding genes in* F. oxysporum *genome*

The *in silico* analyses of putative genes encoding for Cu transporters revealed the existence of six orthologs to *Aspergillu*s genes in *F. oxysporum* (FOXG_03101, FOXG_07770, FOXG_01107, FOXG_01748, FOXG_11217, and FOXG_03265) that share high similarity, between 43.9% and 57.8% of identity (Suppl. Table 1). The *F. oxysporum* FOXG_03265 (XP_018237293.1) (CrpF) displayed 43.9% of identity to Cu-exporting ATPase CrpA (AFUA_3G12740, XP_754347.1) (Figure 2(a)).

**Figure 1.**
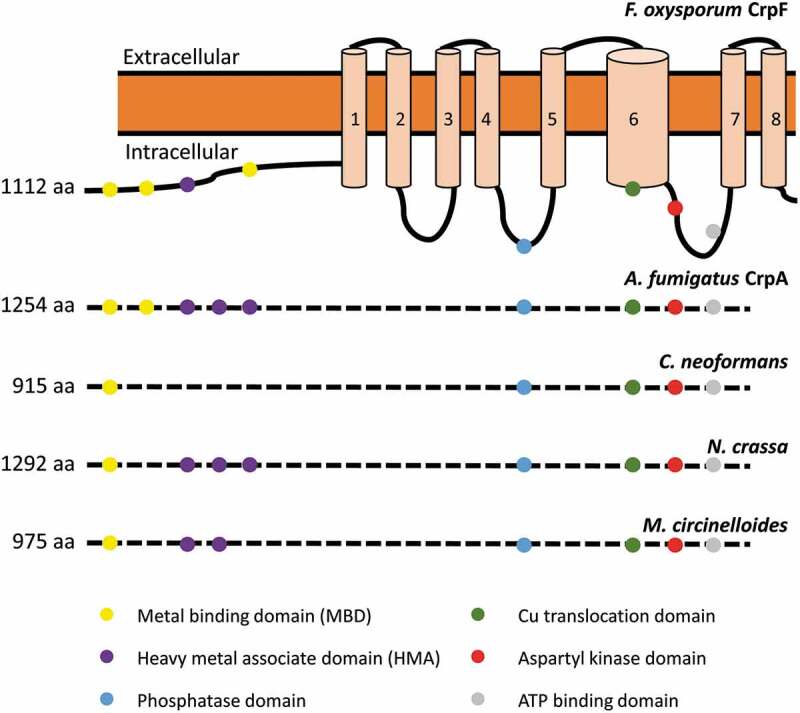
Sequence analysis of the Cu transporter CrpF. Proposed two-dimensional model of CrpF (FOXG_03265; XP_ 018237293.1) protein describing predicted metal-binding motifs (metal-binding domains or heavy metal associated domains), conserved functional domains (phosphatase domain, Cu translocation domain, aspartyl kinase domain, ATP binding domain) and structural domains (8 transmembrane domains) and comparison of the conserved functional domains among different species. GenBank accession numbers are given in parentheses: *A. fumigatus* CrpA (AFUA_3G12740; XP_754347.1), *Cryptococcus neoformans* (OXG38219.1), *Neurospora crassa* (XP_957691.3) and *Mucor circinelloides* (OAD01460.1).

The sequence analyses of FOXG_03265 (XP_018237293.1), designated CrpF, revealed a conserved CPC (Cys-Pro-Cys) Cu translocation motif located in the 6th transmembrane (TM) domain, 4 Cys rich metal-binding motifs (MBD) in the cytoplasmic N-terminal; 3 CxxC motifs located closer to the amino terminus followed by GMxCxxC classical heavy metal associated domains (HMA) (Figure 1). All these domains have been previously described in Cu-transporting ATPases of yeast, human, bacteria, and archaea [18,20,21,40–45]. In common with non-heavy metal-transporting P-type ATPases, CrpF shares characteristic features as an aspartyl kinase domain (DKTG), a phosphatase domain and a consensus domain for ATP binding and energy transduction (GDGINDSP) [41,44]. Fungal Crp-type proteins differ in the number of metal-binding domains (MBD) and heavy metal associated domains (HMA). For instance, Crp in *F. oxysporum* has three MBD and one HMA and *N. crassa* has one MBD and three HMA, whereas *A. fumigatus* has two MBD and three HMA (Figure 1).

**Figure 2.**
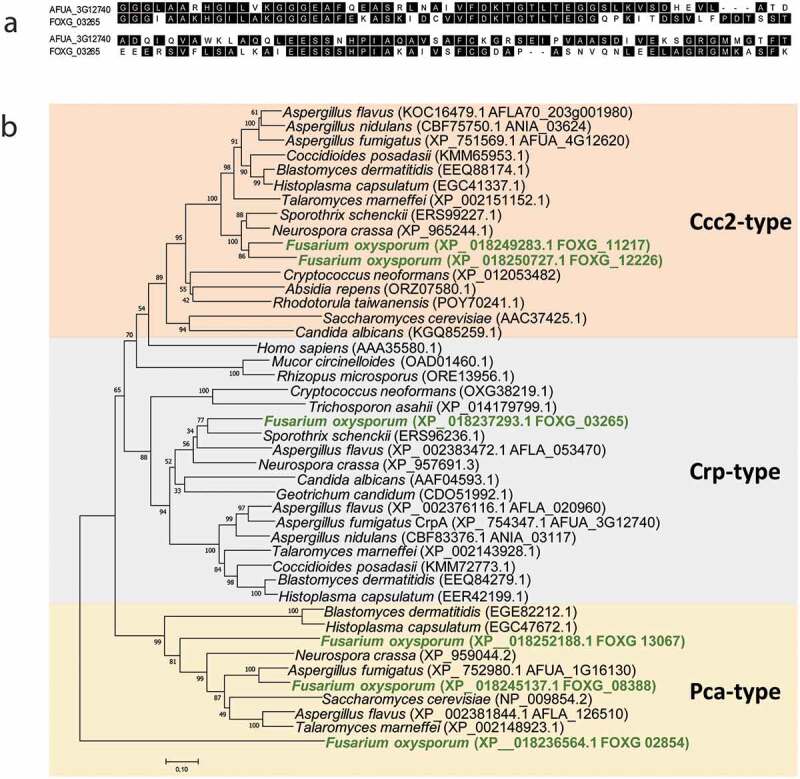
*F. oxysporum* contains a putative Cu-exporting ATPase in its genome. (a) Alignment of the amino acid sequences of the predicted Cu-exporting ATPases encoded by gene FOXG_ 03265 (CrpF) and the corresponding orthologous gene *crpA* from *A. fumigatus* using Clustal method. Protein accession numbers are reported as follows: *A. fumigatus* CrpA (AFUA_3G12740) and *F. oxysporum* CrpF (FOXG_03265). (b) Phylogenetic tree of predicted Heavy-metal ATPases (HMA) in fungal species distributed in clades Cc2-type, Crp-type and Pca-type. The phylogenetic tree depicts clades of HMA protein sequences available at NCBI (sequences ID are shown in parenthesis). HMA proteins are highly conserved among fungi kingdom. In green are highlighted the *F. oxysporum* proteins. Human orthologous gene AAA35580.1 is also included. Phylogram was constructed using Neighbour-Joining method. Bootstrap values obtained from 1000 replicates are indicated at the nodes. Scale bar indicates the relative length of each branch. Clustal W was used for protein alignment.

In order to investigate the conservation degree of P_1B_ type heavy-metal ATPases (HMA) in fungi, a BLASTp analysis was performed. According to phylogenetic analyses and homology searches in fungal genomes using as query the *F. oxysporum* HMA protein orthologous to the PcaA, CrpA, and CptA (Ccc2) in *A. fumigatus*, we found that all *F. oxysporum* sequences were closely clustered within these three divisions (Figure 2(b)). The orthologue species included important plant pathogens, as *Rhizopus microspores*, and human opportunistic fungal pathogens as *C. albicans*.

The Pca-type group derives its name from the *Saccharomyces cerevisiae* Cd transporting ATPase Pca1p [46], the Ccc2-type group was named based on the *S. cerevisiae* Cu-transporting ATPase of the trans-Golgi network Ccc2p [47] and the Crp-type clade derives its name from the *C. albicans* Crp1, a P‐type ATPase plasma membrane protein [22,42].

*F. oxysporum* genome contains six putative genes coding for HMA proteins; one hypothetical protein for Crp-type (FOXG_03265), three for Pca-type (FOXG_13067, FOXG_08388 and FOXG_02854), and two for Ccc2-type clades (FOXG_11217 and FOXG_12226). However, *A. fumigatus* have one protein for each Crp-, Pca- and Ccc2-type clades, respectively (AFUA_3G12740, AFUA_1G16130 and AFUA_4G12620).

In the Crp clade of Cu-exporting ATPases, *A. flavus* was shown to be the unique fungus with two predicted proteins, CrpA (AFLA_020960) and CrpB (AFLA_053470) [23]. Humans contain two Cu ATPase’s, hATP7A (MNK, Menkes disease protein) and hATP7B (WND, Wilson disease protein), both of which carry out Cu delivering and detoxification functions; hATP7A (AAA35580.1) is included in our phylogenetic analysis.

Heavy-metal ATPases (HMA) are highly conserved in pathogenic fungi. Broadly, this is indicative of the widespread distribution of HMA proteins in the evolution among plant pathogenic fungi and human opportunistic fungi pathogens.

### *Target deletion of* crpF *gene*

**Figure 3.**
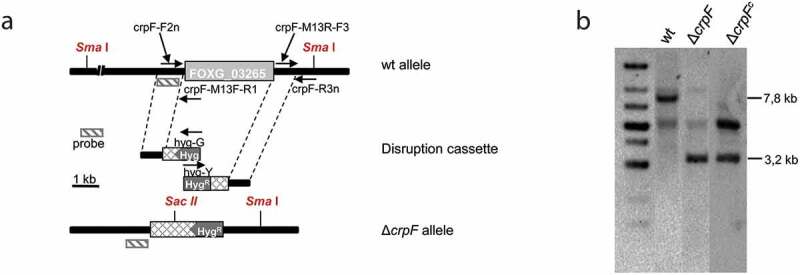
Targeted deletion of the *F. oxysporum crpF* gene. (a) Targeted gene replacement strategy using a disruption construct obtained by fusion PCR with Hyg^R^ cassette as selective marker. Relative positions of primers used for PCR and probe (dashes bars) are indicated. (b) Southern blot analysis of gDNA from wt strain and transformants. gDNAs were digested with *Sma* I and *Sac* II to detect deletion and complementation of *crpF*. The Southern blot image provided comes from two nitrocellulose membranes. The images were manipulated with the objective of only show the interesting transformants.

To investigate the role of *crpF* gene in Cu tolerance and pathogenicity in *F. oxysporum*, a knockout mutant (Δ*crpF*) was generated by targeted gene replacement. Southern analyses of the gDNA from wt and obtained transformants showed a 7.8 kb *Sma* I hybridizing band in wt strain, that was replaced by a 3.2 kb fragment in the homologous transformant (Δ*crpF*#33), indicating the deletion of *crpF* gene sequence (Figure 3(a,b)). Complementation of the Δ*crpF* was performed by cotransformation with the *crpF* wt allele and the Nat^R^ cassette as selective marker. Cotransformants were identified by Southern analyses of gDNA digested with *Sac* II and *Sma* I. Complementation was confirmed on transformant Δ*crpF*^c^#7, which displayed the 3.2 kb *Sac* II/*Sma* I mutant hybridizing band plus an additional band (approximately 6 kb) demonstrating an ectopic integration event (Figure 3(b)). The original Southern blot images are provided in the Suppl. Figure 2.

### F. oxysporum *CrpF contributes to Cu tolerance and increases its accumulation under Cu excess*

The role of CrpF in *F. oxysporum* heavy-metal tolerance was investigated through colony-growth experiments and evaluation of metal sensitivity on wt, knockout mutant (Δ*crpF*) and complemented strain (Δ*crpF*^c^). The ability to tolerate Cd, Cu, and Zn was assessed by determining the minimal inhibitory concentration (MIC value) to each heavy metal in SM media supplemented with different heavy metals. Δ*crpF* showed lower MIC to CdCl_2_ (0.15 mM), CuSO_4_ (0.4 mM) and ZnCl_2_ (20 mM) compared to wt (Suppl. Figure 1).

**Figure 4.**
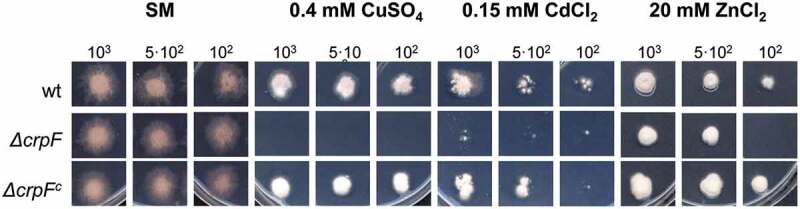
Loss of *crpF* impairs growth of *F. oxysporum* under Cd, Cu and Zn exposure. Fungal colonies from wt, Δ*crpF* and complemented Δ*crpF^c^* strains grown for 6–7 days at 28°C on SM plates containing 0.15 mM Cd (CdCl_2_), 0.4 mM Cu (CuSO_4_) or 20 mM Zn (ZnCl_2_). The number of inoculated spores is indicated.

To better assess growth inhibition effect by metals in wt, ∆*crpF* and ∆*crpF*^c^ strains, we evaluated growth responses (size, shape, and density) at different Cu concentrations. As shown in Figure 4, the colony growth of ∆*crpF* strain showed extreme reduced tolerance to 0.4 mM CuSO_4_ and also higher sensitivity to 0.15 mM CdCl_2_ and to a lesser extend to 20 mM ZnCl_2_. Specifically, Δ*crpF* exhibited a significantly lower cellular density in the central region of the colony in 0.15 mM CdCl_2_ and 20 mM ZnCl_2_.

Our results indicate that CrpF is involved in resistance to Cu toxicity and to a lesser extent to Cd and Zn. Nevertheless, based on our results, we suggest that Cu is the principal substrate of the CrpF; however, Cd or Zn can also be transported. Similarly, the Cu-exporting ATPase CrpA of *A. nidulans* presents other metal substrates besides the principal Cu, such as silver [20]. Interestingly, mycelia cultured under Cd exposure showed colour changes, acquiring orange tones. Some authors have reported the production of orange-brown pigments in fungal colonies, probably due to the induction of pigments that contribute to Cd biosorption onto the cell walls [48,49]. In the case of ZnCl_2_, the mycelia became thicker in its presence compared to the control. This phenomenon of mycelial thickening has also been described in other filamentous fungi [50].

To determine whether hypersensitivity to toxic Cu may be associated to changes in intracellular Cu concentrations in Δ*crpF*, we quantified intracellular Cu by a colorimetric assay that allows to measure the amount of Cu bounds to a specific substrate (BCA) [36].

**Figure 5.**
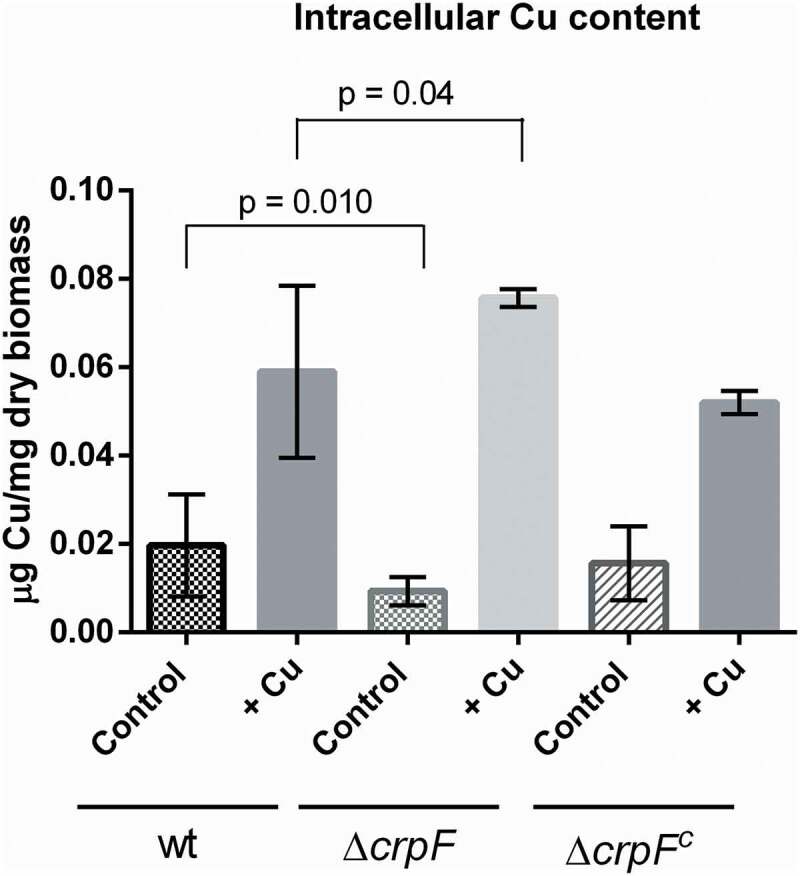
Intracellular Cu determination. Cu concentration was quantified on mycelia from wt and Δ*crpF* strains grown in SM (control) and SM supplemented with 0.1 mM CuSO_4_. The amount of Cu is reported as mg g^−1^ biomass. Values are the average of three replicates.

Quantification of intracellular Cu was performed in mycelia grown during 6 hours with and without Cu addition. The results showed that ∆*crpF* accumulate less Cu (0.009 µg Cu/mg dry biomass) than wt strain (0.020 µg Cu/mg dry biomass) in control conditions (without the addition of Cu) (p = 0.010) (Figure 5). Nevertheless, addition of 0.1 mM CuSO_4_ caused an increase of Cu content in both strains, ∆*crpF* exhibited higher Cu accumulation (0.076 µg Cu/mg dry biomass) than wt (0.058 µg Cu/mg dry biomass) with significant differences (p = 0.04) (Figure 5). Complemented strain ∆*crpF^c^* showed similar Cu accumulation than wt, in both control and metal conditions.

### *Expression pattern of* crpF *in response to metal toxicity*

**Figure 6.**
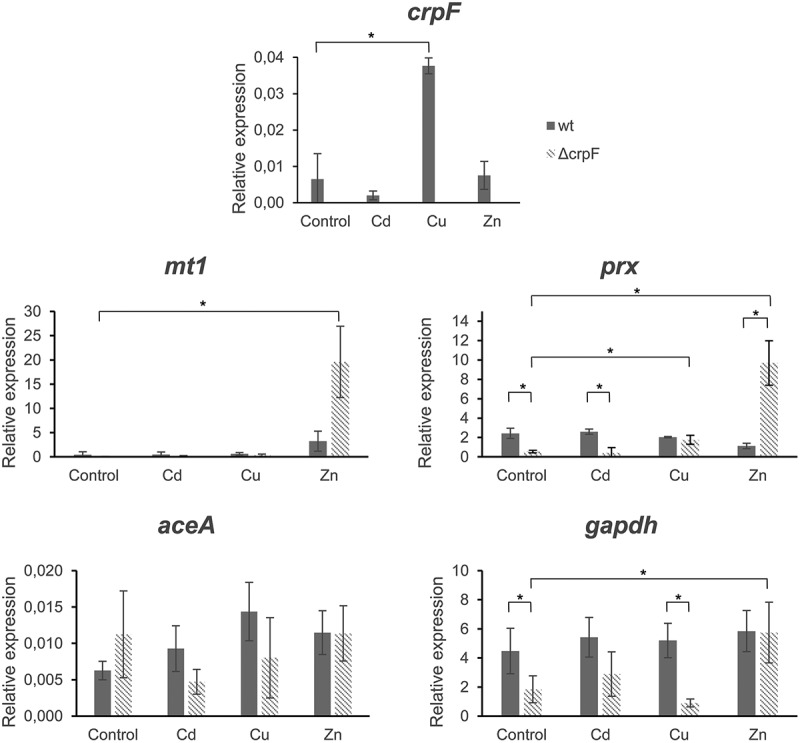
Transcriptional analyses of *crpF*, metal homeostasis *mt1* and *aceA* and stress *prx* and *gapdh* related genes by RT-PCR analysis. Transcript levels of *act, mt1, crpF, aceA, prx* and *gapdh* from wt and Δ*crpF* strains on control condition and under exposure to 0.1 mM CdCl_2,_ 0.175 mM CuSO_4_ or 7.5 mM ZnCl_2_ are shown.

To investigate whether *crpF* is constitutively expressed or induced by metal ions, we determined the expression profile of *crpF* grown in the presence of different heavy metals. Increased *crpF*-transcript levels were detected in the wt strain in the presence of Cu (p = 0.0015), but not in Cd or Zn (p > 0.33). As expected, *crpF* was not transcribed in the Δ*crpF* mutant (Figure 6). To gain a better insight into metal response, we analysed other key genes involved in metal homeostasis and stress response in fungi. The expression of *mt1* (coding for a hypothetical metallothionein), *aceA* (coding for transcription factor), *prx* (coding for peroxiredoxin with antioxidative activity), and *gapdh* (coding for glyceraldehyde-3-phosphate dehydrogenase known to act as oxidative sensor) was determined.

Under control, Cd and Cu conditions, wt and mutant strains displayed different transcript levels of *aceA*, although there were not statistically significant differences. The *aceA* expression in Δ*crpF* was up-regulated in control (p = 0.22) and down-regulated in Cd (p = 0.11) and Cu (p = 0.18). On the other hand, Zn potently induces *mt1* in Δ*crpF* (p = 0.0029) and this overexpression being much greater in the mutant than in the wt (p = 0.0063).

Regarding stress response, upregulation of *gapdh* under Zn exposure but not in the presence of other metals was detected in wt and Δ*crpF* strains (p = 0.044 and p = 0.013, respectively). In general, Δ*crpF* showed slightly lower transcript levels of *gapdh* than wt in all conditions except in presence of Zn, especially with statistical significance in control (p = 0.018) and Cu (p = 0.0025). Another marked difference observed in the mutant is the overexpression of the antioxidant gene *prx* under Zn exposure in comparison with control conditions (p = 0.008) or to wt strain (p = 0.006). Although Δ*crpF* showed lower expression levels than wt of *prx* in the control (p = 0.010) and Cd (p = 0.0009) conditions.

### *The lack of* crpF *does not reduce the ability of* F. oxysporum *to infect tomato plants nor affects resistance to macrophage killing, but increases virulence in mice infection*

**Figure 7.**
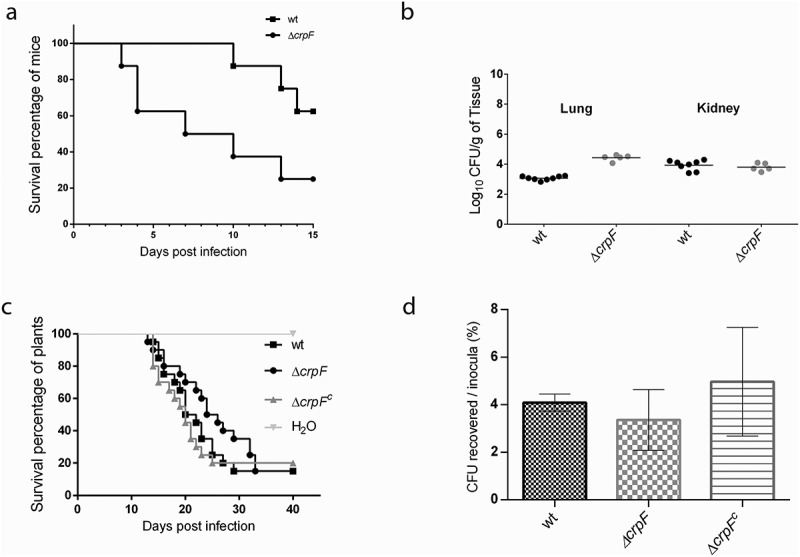
Pathogenic behaviour of Δc*rpF* in different infection models. (a) Virulence of *F. oxysporum* strains in immunosuppressed murine model host inoculated with 10^7^ microconidia of the indicated strains by lateral tail vein injection. Percentage survival values were plotted for 15 days. (b) Fungal load quantification of lung and kidney from eight randomly chosen surviving mice from each strain at day 7 after infection using CFU estimation method. (c) Gene *crpF* is not required for virulence of *F. oxysporum* in a plant model of infection. Survival of groups of 10 tomato plants inoculated by immersing the roots into a suspension of 5 × 10^6^ freshly obtained microconidia mL^−1^ of the indicated strains. Experiments were performed at least three times with similar results and the percentage survival values were plotted for 30 days from one representative experiment. (d) Percentage of viable hypha recovered after 6 h of co-cultivation with macrophages. Data are means of results from duplicated samples from three independent experiments.

The lack of *crpF* does not alter the pathogenic capacity of *F. oxysporum* in tomato plants (Figure 7(c)). When tomato roots were inoculated with microconidial suspensions of the different fungal strains development of vascular wilt symptoms and mortality was similar in plants infected with the Δ*crpF* mutant versus those infected with the wt or the Δ*crpF^c^* (p > 0.05) (Figure 7(c)).

In the macrophage infection not relevant differences between Δ*crpF* and wt were detected, as Δ*crpF* did not display lower resistance to macrophage killing comparing to wt strain (p = 0.23) (Figure 7(d)). Quantification of surviving phagocytized conidia after 6 h of infection showed a 3.3% of viable CFU of ∆*crpF*, a similar value to that detected in the wt (4.1%).

Considering that *F. oxysporum* wt strain can also infect and kill immunosuppressed mice [24], we studied if the lack of *crpF* is essential for infection in a mammalian host. Mortality rates of immunosuppressed mice after challenge with 1 × 10^7^ CFU/animal with wt and Δ*crpF* strains resulted in 37.5% and 75%, respectively (Figure 7(a)). Surprisingly, the mutant was more virulent than the wt strain in mice infection with significant differences (p = 0.0324). The fungal burden in lung was higher in ∆*crpF* versus wt at 1 × 10^7^ CFU/animal (p = 0.0016) (Figure 7(b)). However, no differences in mortality rates among strains were observed when infected with higher inocula (2 x 10^7^ CFU/animal) (data not shown). All these results suggest that *F. oxysporum* CrpF is dispensable for full virulence in plants and mammalian host.

## Discussion

Cu is one of the main essential trace metals required as micronutrient for growth and proliferation in all biological systems. In fungi, both Cu deficiency and overload can be harmful, leading to the miss-regulation of metabolic processes and the alteration of intracellular redox potential. However, organisms have developed mechanisms to adapt to metal fluctuations and to maintain ion homeostatic balance.

Maintaining Cu homeostasis is critical for fungal survival and pathogenesis, and therefore fungi have evolved sophisticated machinery to regulate Cu homeostasis to survive under host-imposed toxicity. Recently, some strategies based on inhibiting the regulators of Cu homeostasis have emerged as an opportunity to improve therapeutic options for aspergillosis [14,15] expanding therapeutic and phytosanitary strategies. Another strategy to create a hostile antimicrobial environment is through antifungal agents that function as Cu ionophores, including 8-hydroxyquinoline (8HQ), Zn pyrithione (ZPT) or boronic ester-masked 8-hydroxyquinoline derivative (QBP) [15]. Cu ionophores can coordinate and translocate Cu from the extracellular environment to the intracellular space. Recently, QBP has been used as antifungal agent to inhibit growth of *C. neoformans* in lungs [14]. Cu tolerance is a critical point for fungal pathogenesis since host cells can inhibit microbial growth by Cu poisoning. Accordingly, it has been proposed that pharmacological disruption of Cu resistance genes, such as *crpA*, could lead to promising strategies for the development of new antifungal therapies. [15]. It is remarkable that the *A. fumigatus* Cu-exporting ATPase CrpA has cysteine-rich Cu-binding motifs (MBD) in its N-terminus, which are not present in the human homolog, which could significantly increase the hypothetical efficacy of that antifungal therapeutic target [15].

Considering the importance of metal ions, Cu uptake and export systems are expected to have a strong impact in fungal resistance and virulence. To reach a new understanding of Cu as a possible virulence factor in human fungal pathogens, we have focused this study on the opportunistic pathogen *F. oxysporum*. For this purpose, we have conducted an *in silico* search in the *F. oxysporum* genome, which has allowed to identify a total of six genes coding for hypothetical Cu transporters orthologs to *S. cerevisiae* and *A. fumigatus*. We have focused on CrpF, the putative homologue of the Cu-exporting ATPase *A. fumigatus* (CrpA, AfuA_3G12740) involved in Cu tolerance and virulence. The importance of this gene is reflected in the high conservation of this metal transporter and genetic duplications. Particularly mutations in the human homologues *crp* genes ATP7A and ATP7B cause X-linked Menkes disease and autosomal recessive Wilson’s disease, respectively [51]. Both genetic disorders are related with a defective Cu homeostasis, leading to Cu deficiency in Menkes disease and toxicity through hepatic Cu accumulation in Wilson’s disease [52].

Cu transporters with high similarity to CrpF have been well described in many fungal organisms in addition to *A. fumigatus*, as *C. albicans* (CaCrp1p, AAF78958.1), *A. nidulans* (CrpA, CBF83376.1) and *A. flavus* (AFLA_020960, AFLA_053470). All characterized Crp proteins from filamentous fungi display the following conserved domains: i) eight transmembrane helix (TMH) that contains the highly conserved CPC [53] motif within the TMH_6_ characteristic for the Cu-ATPases, ii) metal-binding domains (MBD) conformed by speciﬁc Cu-binding sequence located within the NH_2_-terminal containing the consensus CxxC motifs and iii) other heavy metal associated domains (HMA) containing the consensus GMxCxxC [20,54]. Particularly, the *F. oxysporum* CrpF exhibits high identity to the respective *Aspergillus* sp. orthologs, ranging from 42.3% to 47.3% degrees.

Although the presence of metal-binding domains indicates the metallo-regulator activity of metal transporters, more experimental evidences should clarify their metal ion specificity, since many metal transporters show a wide range of metal specificity [1,55].

There are fungal species with better metal tolerance rates, among them *F. oxysporum* has notable tolerance to Cu in synthetic medium (MIC = 1.5 mM CuSO_4_), similar to *S. cerevisiae* (MIC = 2 mM CuSO_4_), or *C. albicans* with a more extreme phenotype (MIC = 20 mM CuSO_4_) [22]. As described in other fungal species, deletion of Cu-exporter genes reduce Cu tolerance [17,22,23,56]. *F. oxysporum* Δ*crpF* mutant showed a severe sensitivity to Cu^2+^. Nonetheless, tolerance to Cu shown by *crp* mutants varies in different species. Thus, the MIC values of CuSO_4_ in *F. osysporum* ∆*crpF* (400 µM) and *C. albicans* Ca*crp1*∆/Ca*crp1*∆ (500 µM) considerably exceeds the MIC of *A. fumigatus* ∆*crpA* (50–150 µM) [17,22,56].

This reduction of Cu tolerance was also observed in the deletion mutant on metallothionein *mt1*, although more moderately [25], suggesting that CrpF should be responsible for large-scale Cu efflux, whereas Mt1 scavenges residual Cu in the cytoplasm.

Regarding tolerance to toxic metal, our study is not the first in testing other metals sensibilities than Cu in a defective strain in a Cu-exporting ATPase. In *A. fumigatus*, ∆*crpA* was not only hypersensitive to high concentrations of Cu but also to Zn, whereas *C. albicans* Ca*crp1*Δ/Ca*crp1*Δ strain was equally resistant to Zn^2+^, Cd^2+^, and Ag^+^ as the wild-type strain [17,22].

Besides to Cu, Δ*crpF* also exhibited sensitivity to Cd and Zn, suggesting that there is no substrate specificity in the transport of this metal. Similarly, it was previously described that *A. nidulans* Δ*crpA* mutant showed sensitivity to Cd^2+^ [20].

The ion-specificity of P_1B_-ATPases seems to derive from the identities and positions of residues within the last TM helices (H_4_-H_6_) which conform the metal-binding site(s) (MBS) [45,57]. Even though Cu-exporting ATPases show substrate specificity, they also present certain promiscuous character. This is the case of *C. albicans* Cu Crd1p ATPase which showed a promiscuous character with respect to metal ion transport towards silver and Cd [42]. As well as it occurs in *S. cerevisiae* Pca1p ATPase which is suggested to be involved in Cu and/or iron homeostasis [58,59].

Metal sensing and transcriptional response is also indicative of intrinsic metal specificity. Previous studies have reported that as consequence of metal loading the detoxifying P_IB_-type ATPase activity is enhanced, either by its overexpression or by modifying its subcellular localization [22,42,52,60]. A case in point is the ATPase PcaA from *A. fumigatus*, which is inducible by Cu or Cd and its lack causes deficiency in Cd tolerance [61]. Also, the P_IB_-type ATPase CaCrp1 in *C. albicans* is induced by extracellular Cu, being essential to cope with low Cu environment and conferring resistance to high Cu concentration by ion extrusion [22,42]. In *A. fumigatus*, CrpA is characterized by removing excess metals from intracellular to the extracellular environment, being critical for both Cu and Zn detoxification while ZrcA transporter has a dominant role in Zn detoxification [56]. However, the transcriptional regulation of *crpA* is yet to be clarified, and further studies are required to determine the nature of its induction by metal ions.

Here, we confirmed by transcriptional studies that *crpF* is Cu-inducible. In addition, tolerance assays showed that the lack of *crpF* not only strongly decreases Cu tolerance but also reduce tolerance to Cd and Zn compared to wt. However, neither the presence of Cd nor Zn seems to induce the expression of this metal transporter in *F. oxysporum*, which indicates its Cu sensing specificity.

This expression gene trait also occurred in *A. nidulans* as Cd did not induce *crpA* under low concentrations [20]. The authors concluded that those results may be indicating an indirect P_IB_-type ATPase regulation, possibly due to the saturation of the main Cd detoxification system, a Cd^2+^ pump. Similarly, *F. oxysporum* CrpF mediated heavy-metal detoxification is not restricted to Cu but also to Cd and Zn, despite being a specific Cu-inducible gene. We suggest that Cd is likely a non-inductive substrate of CrpF in *F. oxysporum*, due to a substrate promiscuity of this metal transport [62].

Extended transcriptional analysis of metal-responsive genes or stress-related genes demonstrated that the lack of *crpF* leads to a strong activation of the metallothionein *mt1* and the peroxiredoxin *prx* in the presence of Zn, both related to metal toxicity and oxidative stress, respectively [25,63].

We hypothesize that Zn sensing and regulation is tightly controlled in cells, thus toxic concentrations may activate the expression of *mt1* in order to metal-buffering [25] and *prx* to cope with the oxidative stress cause by intracellular Cu excess [35]. An increase in the *prx* expression has been related to antioxidant role against ROS [63,64]. However, in *F. oxysporum prx* overexpression was only observed in the presence of Zn in the Δ*crpF* mutant. This may indicate that *crpF* would be indirectly participating in Zn homeostasis, although its absence has almost no impact on the tolerance to this metal ion in *F. oxysporum.*

It has been suggested that the over-expression of *gapdh* might be a consequence of the reconfiguration of the glycolytic flux, a mechanism reported to regulate the response to oxidative stress in human, plant, and yeast cells [65–67]. It has been observed increased expression of *gapdh* triggered by Cu in *Candida humilis* and in *Staphylococcus aureus* [35,68]. However, in wt strain no difference was observed in *gapdh* expression between control and Cu-enriched conditions. Thus, *F. oxysporum* displays a different regulatory profile in response to heavy-metal stress, compared to other fungi.

In *S. cerevisiae*, Ace1 regulates genes encoding for superoxide dismutase (Sod1) or metallothioneins (Cup1 and Crs5) under metal-replete conditions [69]. In *A. fumigatus*, the orthologous transcription factor AceA is involved in Cu and Zn detoxification through up‐regulation of the transporter, CrpA and ZrcA [17,56]. In *A. nidulans*, AceA is necessary for metal inducible expression of *crpA*, but not of the putative metallothionein *crdA* [20]. In our study, the *aceA*-transcript levels in the presence of toxic metals were slightly higher than in control conditions in *F. oxysporum* wt strain, although without statistically significant differences. Moreover, the deficiency of *crpF* does not increase *aceA* transcription under metal toxic conditions, which may indicate that other transcription factors may be controlling metal stress response in this fungus.

Evidence of the direct participation of CrpF in the Cu transport is supported by the increased accumulation of intracellular Cu in the Δ*crpF* strain under excess Cu exposure. However, the opposite occurs in the absence of metals, where the mutant strain accumulates less Cu. Considering this, the mutant strain differs significantly from the wt, indicating that CrpF contributes to cellular homeostasis as a protective response, exporting Cu when intracellular Cu is elevated or enabling maintenance of Cu levels when is scarce.

Deletion of the Cu-exporting ATPase *crpF* in *F. oxysporum* did not result in virulence attenuation in a murine model of disseminated fusariosis. In contrast, Δ*crpF* showed greater virulence with statistically significant differences between Δ*crpF* and wt in survival rates (p = 0.0324) and in fungal burden in lungs (p = 0.0016). In opposition to the previously reported in other fungal species, in which the lack of a Cu-exporter resulted essential for full fungal virulence, for instance, in *C. albicans*, the Cu-exporting ATPase (Crp1p) resulted essential for establishing full virulence [16]. Similarly, the putative Cu-exporting ATPase CrpA from *A. fumigatus* was necessary for virulence in the non-neutropenic invasive aspergillosis murine model [17].

The fact that in *F. oxysporum* the absence of *crpF* results in increased virulence in mice infections is a controversial result that requires more research on intracellular Cu trafficking. Moreover, our data indicate that Δ*crpF* displayed hardly any deficiency in pathogenic capacity in macrophage infection. The ratio CFU recovered/inoculum was decreased in Δ*crpF* but not significantly, suggesting the participation of other metal transporter proteins in Cu detoxification. Our results differ from those reported on *A. fumigatus*, in which spore survival assays with murine alveolar macrophages showed significantly reduced viability of Δ*crpA* mutant [17].

Similarly, the lack of *crpF* did not affect the virulence of *F. oxysporum* in tomato plant infection, suggesting that *crpF* is not involved in plant wilt disease this unless under the assayed conditions. Despite the fact that Cu-exporting ATPases are essential for a correct balance of metals in fungi, the lack of toxicity by means of metal ions during plant infections can be decisive so that deletion *crpF* does not have impact on its pathogenic capacity. This is in line with other reported studies of Cu-exporting ATPases in other fungi. For instance, in *A. flavus* the Cu-exporting ATPases CrpA and CrpB contribute to virulence in animal infection model, but not in plant infection [23]. Conversely, other fungi require Cu-exporting ATPases to efficiently penetrate and infect plant tissue, as occurs in *Botrytis cinerea* and *Colletotrichum* [70,71].

Our study reflects how *crpF* is essential dispensable gene for fungal virulence in plant or mammalian host, despite being a key gene in metal homeostasis.

In conclusion, this work provides a better understanding of the function of a Cu-exporting ATPase in *F. oxysporum* and helps to complete the picture of the role of the Cu-exporting ATPases in fungal tolerance and pathogenesis.

## Conclusions

We have identified a P_IB_-type ATPase *crpF* gene in the *F. oxysporum* genome that shows the typical structure and domains distribution described for that family of proteins. Particularly, the presence of cysteine-rich metal-binding domains reinforces the hypothesis of its involvement in Cu resistance. The present study provides the first evidence that the *F. oxysporum* Cu-exporting ATPase CrpF contributes significantly to increasing tolerance to Cu, Cd, and Zn, and is not required for fungal pathogenesis in plant or mammalian infection. Interestingly, Δ*crpF* mutant seems to be affected in Cu export, as it undergoes a greater intracellular Cu accumulation than that observed in the wt strain. This metal transporter is a metal-responsive gene since Cu levels regulate its transcription. In addition, the Δ*crpF* response to Zn stress led to enhanced transcription of *prx*, activated usually under cellular oxidative stress. This indicates that Δ*crpF* triggers oxidative stress response in the presence of Zn, possibly due to the activation of mechanisms that would be contributing to metal tolerance.

Regarding pathogenicity, there are still few reports describing the role of P_IB_-type ATPases in fungal virulence. The works reported so far indicate that deletion of *crp* genes in other pathogenic fungi causes decreased fungal virulence in mouse model of invasive infection and plant infections [16,17,23,70,71]. But in *F. oxysporum* the opposite is observed, loss of function of *crpF* increases virulence in a murine model of systemic infection and exhibits similar survival within the macrophages phagosome and plant infection.

In conclusion, our study highlights the role of CrpF in Cu homeostasis, contributing to metal detoxification by transporting Cu excess across membranes. Given that advancing knowledge of the mechanisms involved in fungal Cu homeostasis will be helpful in reaching a better understanding of the fungal infections and host–fungus interactions, we believe that further research is required.

## Supplementary Material

Supplemental Material
