# Supplementary material for: Cu transporter protein CrpF protects against Cu-induced toxicity in Fusarium oxysporum
Source: Virulence. 2020 Aug 30;11(1):1108–21. doi: 10.1080/21505594.2020.1809324 (PMC7549990; doi:10.1080/21505594.2020.1809324)
Supplement: Supplemental Material [file KVIR_A_1809324_SM9592.pptx]

## Slide 1
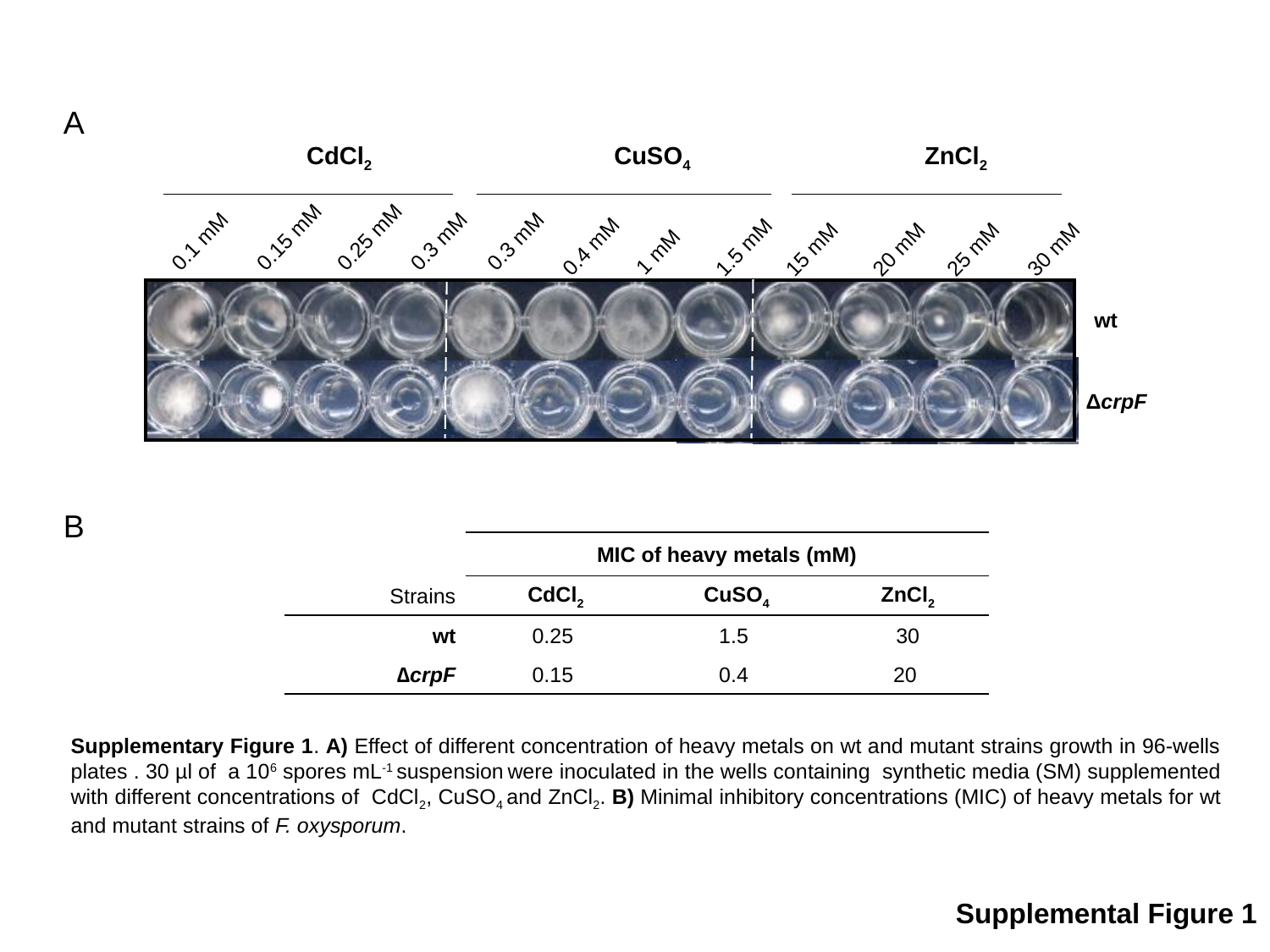

A
CdCl2
CuSO4
ZnCl2
0.1 mM
0.15 mM
0.25 mM
0.3 mM
1.5 mM
15 mM
20 mM
25 mM
30 mM
0.4 mM
0.3 mM
1 mM
wt
ΔcrpF
B
| | MIC of heavy metals (mM) | | |
| --- | --- | --- | --- |
| Strains | CdCl2 | CuSO4 | ZnCl2 |
| wt | 0.25 | 1.5 | 30 |
| ∆crpF | 0.15 | 0.4 | 20 |
Supplementary Figure 1. A) Effect of different concentration of heavy metals on wt and mutant strains growth in 96-wells plates . 30 µl of a 106 spores mL-1 suspension were inoculated in the wells containing synthetic media (SM) supplemented with different concentrations of CdCl2, CuSO4 and ZnCl2. B) Minimal inhibitory concentrations (MIC) of heavy metals for wt and mutant strains of F. oxysporum.
Supplemental Figure 1

## Slide 2
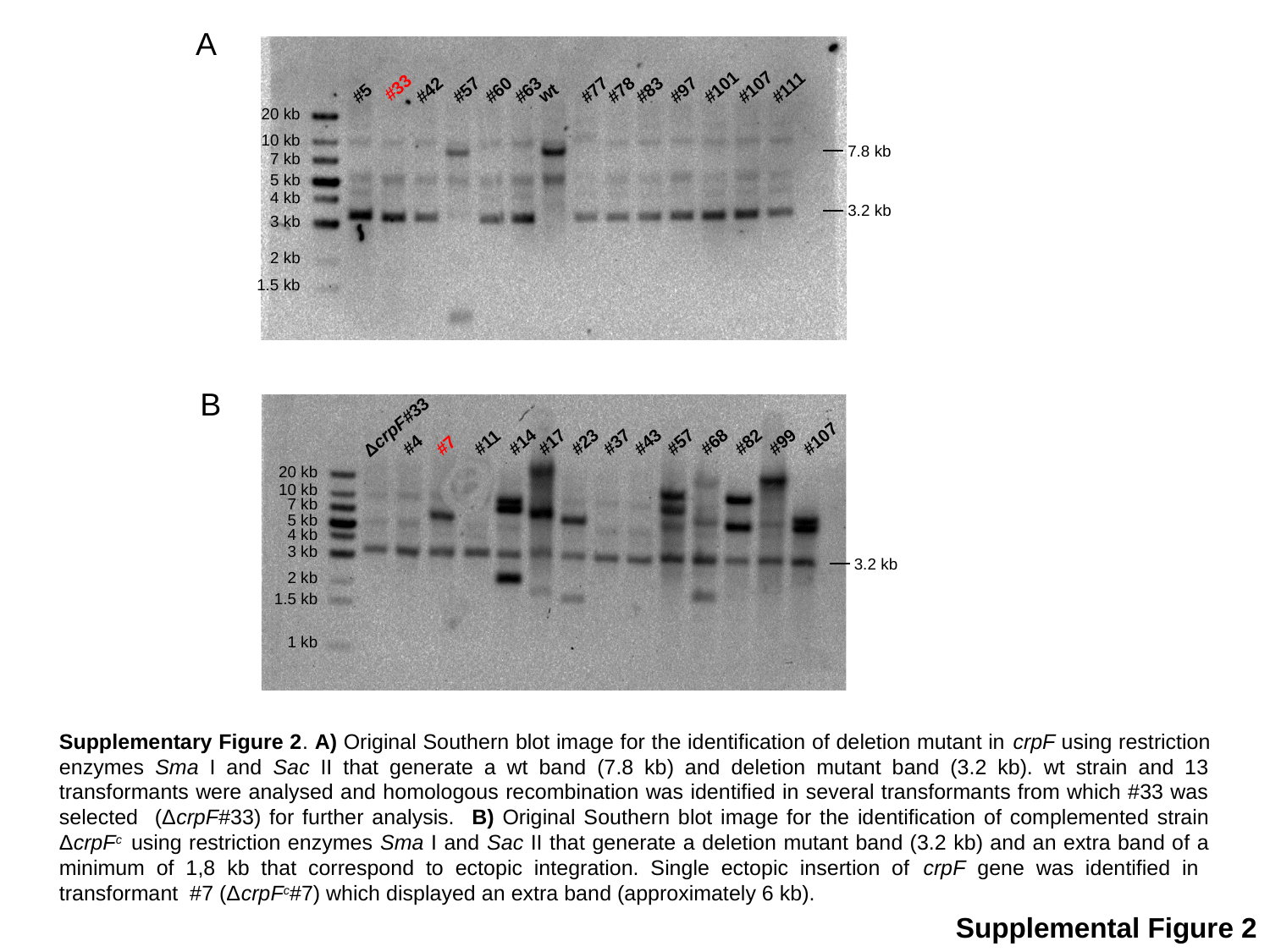

A
#33
wt
#5
#42
#57
#60
#63
#77
#78
#83
#97
#101
#107
#111
20 kb
10 kb
7.8 kb
7 kb
5 kb
4 kb
3.2 kb
3 kb
2 kb
1.5 kb
B
#7
ΔcrpF#33
#4
#23
#11
#14
#17
#37
#43
#57
#68
#82
#99
#107
20 kb
10 kb
7 kb
5 kb
4 kb
3 kb
3.2 kb
2 kb
1.5 kb
1 kb
Supplementary Figure 2. A) Original Southern blot image for the identification of deletion mutant in crpF using restriction enzymes Sma I and Sac II that generate a wt band (7.8 kb) and deletion mutant band (3.2 kb). wt strain and 13 transformants were analysed and homologous recombination was identified in several transformants from which #33 was selected (ΔcrpF#33) for further analysis. B) Original Southern blot image for the identification of complemented strain ΔcrpFc using restriction enzymes Sma I and Sac II that generate a deletion mutant band (3.2 kb) and an extra band of a minimum of 1,8 kb that correspond to ectopic integration. Single ectopic insertion of crpF gene was identified in transformant #7 (ΔcrpFc#7) which displayed an extra band (approximately 6 kb).
Supplemental Figure 2

## Slide 3
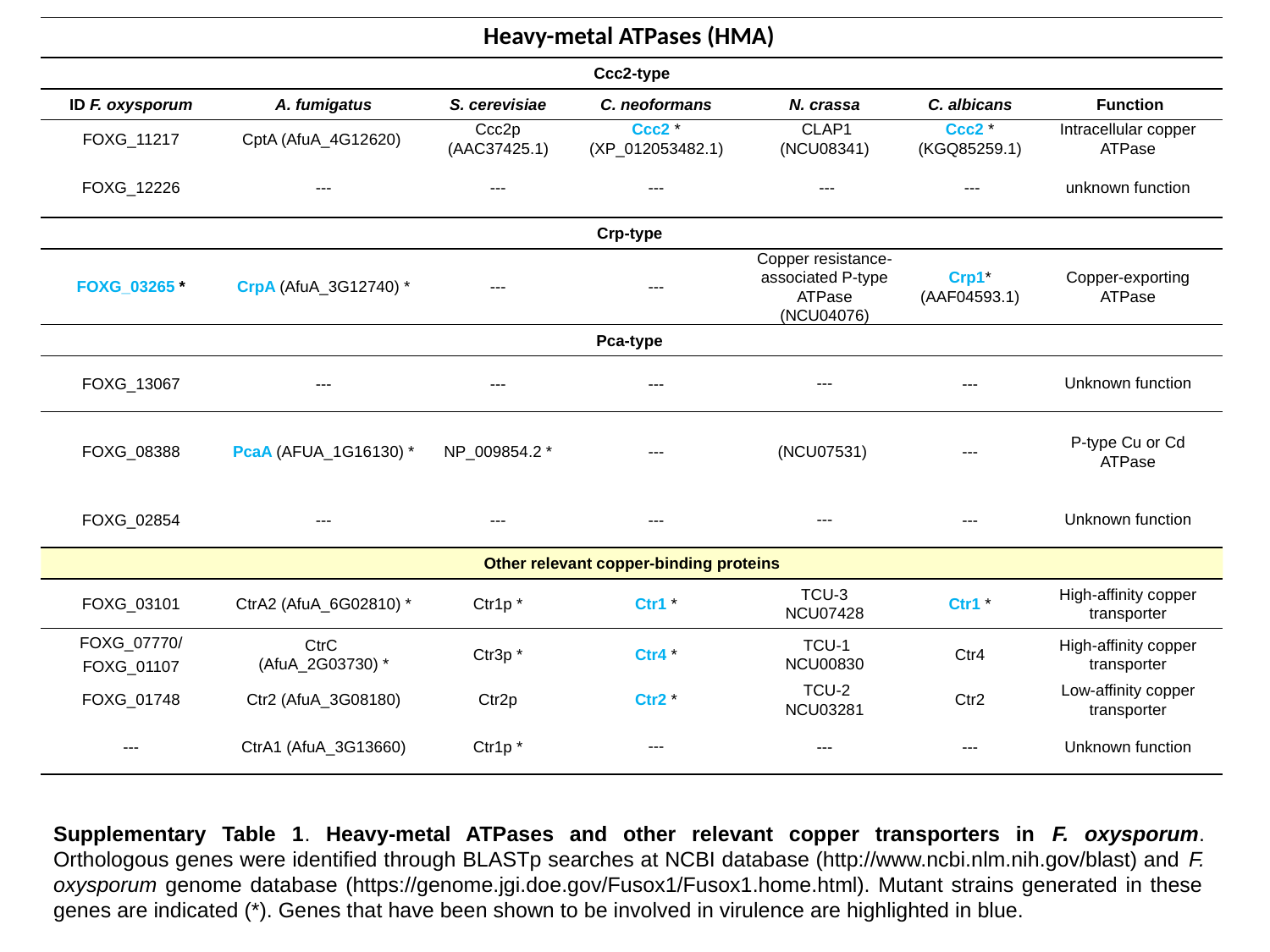

| Heavy-metal ATPases (HMA) | | | | | | |
| --- | --- | --- | --- | --- | --- | --- |
| Ccc2-type | | | | | | |
| ID F. oxysporum | A. fumigatus | S. cerevisiae | C. neoformans | N. crassa | C. albicans | Function |
| FOXG\_11217 | CptA (AfuA\_4G12620) | Ccc2p (AAC37425.1) | Ccc2 \* (XP\_012053482.1) | CLAP1 (NCU08341) | Ccc2 \* (KGQ85259.1) | Intracellular copper ATPase |
| FOXG\_12226 | --- | --- | --- | --- | --- | unknown function |
| Crp-type | | | | | | |
| FOXG\_03265 \* | CrpA (AfuA\_3G12740) \* | --- | --- | Copper resistance-associated P-type ATPase (NCU04076) | Crp1\* (AAF04593.1) | Copper-exporting ATPase |
| Pca-type | | | | | | |
| FOXG\_13067 | --- | --- | --- | --- | --- | Unknown function |
| FOXG\_08388 | PcaA (AFUA\_1G16130) \* | NP\_009854.2 \* | --- | (NCU07531) | --- | P-type Cu or Cd ATPase |
| FOXG\_02854 | --- | --- | --- | --- | --- | Unknown function |
| Other relevant copper-binding proteins | | | | | | |
| FOXG\_03101 | CtrA2 (AfuA\_6G02810) \* | Ctr1p \* | Ctr1 \* | TCU-3 NCU07428 | Ctr1 \* | High-affinity copper transporter |
| FOXG\_07770/ FOXG\_01107 | CtrC (AfuA\_2G03730) \* | Ctr3p \* | Ctr4 \* | TCU-1 NCU00830 | Ctr4 | High-affinity copper transporter |
| FOXG\_01748 | Ctr2 (AfuA\_3G08180) | Ctr2p | Ctr2 \* | TCU-2 NCU03281 | Ctr2 | Low-affinity copper transporter |
| --- | CtrA1 (AfuA\_3G13660) | Ctr1p \* | --- | --- | --- | Unknown function |
Supplementary Table 1. Heavy-metal ATPases and other relevant copper transporters in F. oxysporum. Orthologous genes were identified through BLASTp searches at NCBI database (http://www.ncbi.nlm.nih.gov/blast) and F. oxysporum genome database (https://genome.jgi.doe.gov/Fusox1/Fusox1.home.html). Mutant strains generated in these genes are indicated (*). Genes that have been shown to be involved in virulence are highlighted in blue.
